# Inhibition of KDM2/7 Promotes Notochordal Differentiation of hiPSCs

**DOI:** 10.3390/cells13171482

**Published:** 2024-09-04

**Authors:** Martha E. Diaz-Hernandez, Kimihide Murakami, Shizumasa Murata, Nazir M. Khan, Sreekala P. V. Shenoy, Katrin Henke, Hiroshi Yamada, Hicham Drissi

**Affiliations:** 1 Department of Orthopaedics, Emory University, Atlanta, GA 30329, USA; martha.elena.diaz.hernandez@emory.edu (M.E.D.-H.); km042087@wakayama-med.ac.jp (K.M.); shizuman@wakayama-med.ac.jp (S.M.); mohd.n.khan@emory.edu (N.M.K.); sshenoy@wakehealth.edu (S.P.V.S.);; 2 Atlanta VA Medical Center, Decatur, GA 30033, USA; 3 Department of Orthopaedics Surgery, Wakayama Medical University, Wakayama 641-8510, Japan; yamacha@wakayama-med.ac.jp

**Keywords:** human iPSC, notochordal cells, intervertebral disc degeneration, epigenetics, bulk RNA transcriptomics, differentiation, KDM

## Abstract

Intervertebral disc disease (IDD) is a debilitating spine condition that can be caused by intervertebral disc (IVD) damage which progresses towards IVD degeneration and dysfunction. Recently, human pluripotent stem cells (hPSCs) were recognized as a valuable resource for cell-based regenerative medicine in skeletal diseases. Therefore, adult somatic cells reprogrammed into human induced pluripotent stem cells (hiPSCs) represent an attractive cell source for the derivation of notochordal-like cells (NCs) as a first step towards the development of a regenerative therapy for IDD. Utilizing a differentiation method involving treatment with a four-factor cocktail targeting the BMP, FGF, retinoic acid, and Wnt signaling pathways, we differentiate CRISPR/Cas9-generated mCherry-reporter knock-in hiPSCs into notochordal-like cells. Comprehensive analysis of transcriptomic changes throughout the differentiation process identified regulation of histone methylation as a pivotal driver facilitating the differentiation of hiPSCs into notochordal-like cells. We further provide evidence that specific inhibition of histone demethylases KDM2A and KDM7A/B enhanced the lineage commitment of hiPSCs towards notochordal-like cells. Our results suggest that inhibition of KDMs could be leveraged to alter the epigenetic landscape of hiPSCs to control notochord-specific gene expression. Thus, our study highlights the importance of epigenetic regulators in stem cell-based regenerative approaches for the treatment of disc degeneration.

## 1. Introduction

Intervertebral disc disease (IDD) is a debilitating condition that can be caused by intervertebral disc (IVD) damage which progresses towards deterioration and dysfunction resulting in spine instability. IDD affects more than 400 million individuals globally, thus representing an economic burden worldwide [1]. The disc degenerative process mostly initiates at the nucleus pulposus (NP) level due to the reduced ability of NP cells to regenerate and produce proteoglycans after damage. Cell-based regenerative therapy provides a promising strategy for restoring and repairing degenerated discs [2,3,4]. Recently, human pluripotent stem cells (hPSCs) were recognized as an attractive source for regenerative medicine in skeletal diseases [5]. In this context, emerging methodologies for deriving NP cells are based on the known molecular signaling mechanisms involved in the formation of the embryonic origin of IVD cells [6]. In a recent study, we demonstrated that notochordal-like cells (NCs), which are progenitors of the NP, can be derived from human embryonic stem cells (hESCs) [7]. However, ethical considerations regarding the utilization of hESCs present a notable barrier. Therefore, adult somatic cells reprogrammed into human induced pluripotent stem cells (hiPSCs) represent an attractive alternative cell source for the derivation of NC cells. Nonetheless, fundamental questions about terminal differentiation potential, cell survival, differentiation efficiency, and cell recovery yield from current or new methodologies need to be explored before they can be used for cell replacement therapy. 

Epigenetic regulation has recently been recognized as a key player in the lineage-specific differentiation of iPSCs [8,9,10]. Among epigenetic regulatory mechanisms, histone modification is the most studied method of chromatin regulation which usually occurs at lysine and arginine residues. Histone methylation alters histone–DNA binding affinity and thus regulates DNA accessibility and gene expression [11]. Histone demethylases are epigenetic enzymes that remove methyl groups from lysine and arginine residues on histones. Several recent studies showed that histone demethylases play an essential regulatory function in the cell fate decisions of MSCs [12]. Further epigenetic studies suggest a role of histone lysine demethylases (KDMs), such as KDM2A/B, KDM4B/C, KDM5A, KDM6A/B, and KDM7A, as osteogenic and adipogenic regulators, mainly through the regulation of expression of key genes during the differentiation process [13,14,15,16,17]. While KDMs are recognized as negative regulators of osteogenic differentiation of MSCs, their role in the differentiation of hiPSCs towards notochordal cells is largely unknown. 

In the present work, we used CRISPR/Cas9-generated mCherry-reporter knock-in hiPSCs as a genetic tool to determine the potential for notochordal-like cell derivation. We utilized sequential treatment with a cocktail of four factors targeting the BMP, FGF, retinoic acid, and Wnt signaling pathways to efficiently differentiate hiPSCs into notochordal-like cells. We performed a comprehensive analysis of transcriptomic changes throughout the differentiation process and identified the regulation of histone methylation as a pivotal driver facilitating the differentiation of hiPSCs into notochordal-like cells. We found that specific inhibition of histone demethylases KDM2A and KDM7A/B enhanced the lineage commitment of hiPSCs towards notochordal-like cells by enhancing the expression of key notochordal marker genes. Our results suggest that inhibition of KDMs could be leveraged to alter the epigenetic landscape of hiPSCs to control notochord-specific gene expression and notochord differentiation for the therapeutic application of disc repair. Our study thus highlighted the importance of epigenetic regulators in stem cell-based regenerative approaches for the treatment of disc degeneration.

## 2. Materials and Methods

### 2.1. Generation of the Noto-2A-mCherry hiPSC Clones

This research article describes a study involving commercial human cell lines. “Patient consent is not applicable.” The human dermal fibroblast line hDFa-YK27 was used to generate human induced pluripotent stem cells (YK27-hiPSCs), which were used for the insertion of the mCherry sequence. The endogenous NOTO gene (accession: NM_001134462.2) was used to generate the Noto-2A-mCherry targeting vector containing the 2A-mCherry sequence after exon 3 and before the 3′UTR sequence of the NOTO gene. We used the CRISPR-Cas9 system to insert the Noto-2A-mCherry reporter vector into YK27-hiPSCs using our previously published strategy on hESCs [7]. Multiple clones were generated, and genomic DNA was used to identify homozygous (#10.1 and #10.4) and heterozygous clones (#14.3 and #14.1). Sanger sequencing was used to verify the presence of NOTO-2A-mCherry in the hiPSC clones. 

### 2.2. hiPSC Subculturing and Maintenance

To avoid clonal bias, three independent hiPSC Noto-2A-mCherry-hiPSC clones were analyzed (#10.1, #14.3, and #10.4). hiPSC colonies were maintained in mTeSR^TM^ Plus media (STEMCELL Technologies, Vancouver, CA, USA) on 0.1% Geltrex-coated plates (Peprotech/Thermo Fisher Scientific, Waltham, MA, USA). The hiPSC colonies were passaged when they reached 80% confluency. For notochordal differentiation experiments, hiPSC clones were passaged in 10 cm plates using ReLeSR solution (STEMCELL Technologies, Vancouver, CA, USA) according to the manufacturer’s protocol. ReLeSR dissociation reagent was carefully monitored to obtain medium-sized cell aggregates. hiPSCs were lifted and plated at 80% cell density and cultures were maintained for 3 days in mTeSR^TM^ Plus media until treatments started.

### 2.3. Immunofluorescence on hiPSC Clones and Pluripotency Markers

Verification of the pluripotency of the parental Noto-2A-mCherry-hiPSC line was performed using Immunofluorescence staining as described previously [18]. In brief, Noto-2A-mCherry-hiPSC lines were cultured in feeder-free conditions in mTeSR^TM^ Plus medium. After reaching 80% confluency, clones were fixed in 4% paraformaldehyde, and permeabilized in 0.1% Triton X-100 PBS. The Pluripotent Stem Cell 4-Marker Immunocytochemistry Kit (Thermo Fischer Scientific) was used to detect SSEA4 and OCT4 protein expression in hiPSC clones. Cells were incubated overnight at 4 °C with antibodies against SSEA4 and OCT4 followed by incubation in Alexa Fluor-488 anti-mouse IgG and Alexa Fluor-555 anti-rabbit secondary antibodies for detection. Nuclei were counterstained with DAPI using Fluoroshield Mounting medium (ab104139, Abcam, Cambridge, UK). Images were acquired using a Lionheart FX Automated Microscope (BioTek Instruments, Winooski, VT, USA).

### 2.4. Derivation of Notochordal-like Cells from hiPSCs

To induce the notochordal cell differentiation of Noto-2A-mCherry-hiPSCs, we modified our previously published protocol for hESCs [7]. Briefly, hiPSCs were cultured on Geltrex-coated 10 cm plates in mTeSR^TM^ Plus for 3 days, and then mTeSR^TM^ Plus media was replaced with basal differentiation media containing IMDM (Gibco/Thermo Fischer Scientific); Ham’s F12 (Gibco/Thermo Fischer Scientific); 150 mM MTG (Sigma-Aldrich, St. Louis MO, USA); 0.5 mM ascorbic acid (Sigma-Aldrich); 1× pen/strep (Gibco/Thermo Fischer Scientific); 0.5% B-27 supplement (Gibco/Thermo Fischer Scientific); 0.5% N2 supplement (Gibco/Thermo Fischer Scientific); and 0.05% BSA (Sigma-Aldrich). We used three experimental groups during the differentiation process: (1) Basal control: hiPSCs cultured in basal differentiation media; (2) LAF group: hiPSCs cultured in basal differentiation media supplemented with an LAF cocktail and collected after 3 days of treatment. The LAF cocktail consisted of 0.75 µM BMP inhibitor **L**DN 193189 **(L)** (Sigma-Aldrich), 1 µM of pan receptor inhibitor of retinoic acid signaling **A**GN193109 **(A)** (Tocris, Bristol, UK), and 10 ng/mL b**F**GF **(F)** (PeproTech/Thermo Fisher Scientific) growth factor; (3) LAFC group: hiPSCs cultured in basal differentiation media supplemented with the LAF cocktail for 3 days followed by addition of 3 µM GSKβ inhibitor **C**HIR99021 (**C**) to the LAF cocktail (LAFC: LAF cocktail + CHIR99021). Cultures were maintained until day 5. Media and factors were changed every alternate day. 

### 2.5. Treatment with Daminozide (iKDM) during Notochordal Differentiation

To assess the role of KDM inhibition on notochordal cell derivation, Daminozide (Cayman, Ann Arbor MI, USA), a small molecule inhibitor of KDM2A, KDM7A, and KDM7B, was used. Experimental groups consist of a cocktail of LAF factors supplemented with iKDM (LAF + iKDM) on the first day of differentiation and maintained for 3 days, and then the culture was supplemented with CHIR99021 (LAFC + iKDM) at day 3 and maintained until day 5 of differentiation.

### 2.6. Fluorescence Microscopy to Visualize mCherry-Positive Notochordal Cells

The notochordal differentiation was monitored on day 3 and day 5 of the differentiation process by visualizing mCherry fluorescence (Texas Red Channel), which denotes the expression of the NOTO gene. Visualization of mCherry fluorescence was performed using a Lionheart FX Automated Microscope (BioTek Instruments). The areas with a high intensity of red fluorescence were used as markers for the efficiency of notochordal derivation.

### 2.7. Quantitative Real-Time PCR to Analyze mRNA Expression Levels of Notochordal Marker Genes

Noto-2A-mCherry-hiPSC clones were harvested on days 0, 3, and 5 of notochordal differentiation. Total messenger RNA from isolated cells was prepared using TRIzol™ reagent (Invitrogen/Thermo Fisher Scientific) and cDNA was synthesized from 1μg of total RNA using a Verso cDNA synthesis kit (Thermo Fisher Scientific) for all experimental groups. mRNA expression levels of the pluripotency marker genes (*OCT4*, *NANOG*, and *SOX2*) and notochordal marker genes (*NOTO*, *FOXA2*, *FOXA1*, *SHH*, *T*, *SLIT*, *CHORDIN*, and *AGGRECAN*) were quantified using SYBR^®®^Green assays, and expression levels were calculated using the 2^−ΔΔCT^ method as previously described [7]. *GAPDH* was used as the housekeeping gene and as an internal control.

### 2.8. Bulk RNA Sequencing and Transcriptomic Analyses

To determine the molecular changes during notochordal differentiation, we performed transcriptomic analysis. Total RNA was extracted using miRNeasy Kits (Qiagen, Hilden, Germany). Genomic DNA was removed by On-Column DNase digestion during extraction. The quality of RNA was determined by analyzing the RNA integrity Number (RIN) using an Agilent 2200 Bioanalyzer (Agilent, Alpharetta, GA, USA). Samples having a RIN value > 7 were used for library preparation. Poly A enrichment method was used to prepare the library using TruSeq Stranded Total RNA Sample Prep Kit (Illumina, San Diego, CA, USA). Sequencing was carried out using the NovaSeq PE 150 system (Novogene, Beijing, China). 

High quality, adapter trimmed reads provided by Novogene were mapped to the human reference genome (GRCh38) using STAR (version 2.7.10b) [19]. DESeq2 was used to determine read counts and to perform differential gene expression (DEGs) analysis (version 1.12.3) [20]. Statistically significant DEGs were defined by an adjusted *p* value < 0.05 and log_2_ fold change >2 or <−2. Multiple correction testing was performed using False Discovery Rate (FDR) method. Reads Per Kilobase per Million mapped reads (RPKM) values were calculated using EdgeR (version 3.14.0) [21], and were used for pair-wise comparison between different treatment groups during notochordal differentiation. 

Heatmaps and volcano plots of selected genes were generated using the R Bioconductor package (version 3.19) [22]. Gene Ontology (GO) enrichment analysis was performed using STRING (v11.0) [23]. 

### 2.9. Statistical Analysis

All data are presented as the Mean ± SD, and the statistically significant difference between the experimental groups and controls was analyzed using one-way ANOVA followed by post hoc analyses using the Tukey test (Prism, version 8.1.2, GraphPad Software, La Jolla CA, USA). Unless otherwise noted, each experiment was repeated at least three times using three independent samples. *p* < 0.05 was considered to be statistically significant.

## 3. Results

### 3.1. Generation and Characterization of Notochordal Reporter Pluripotent Stem Cells 

**Figure 1:**
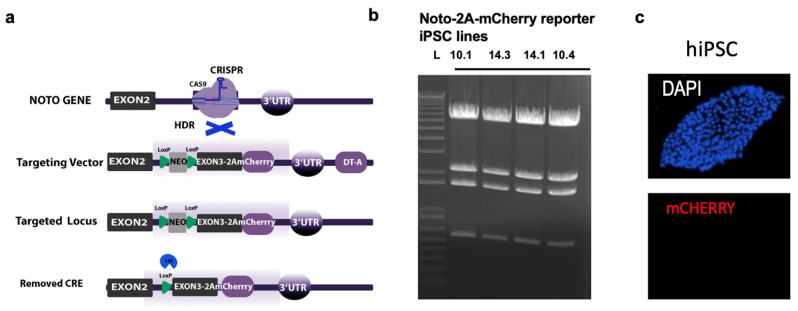
Generation of Noto-2A-mCherry reporter human iPSC clones (hiPSCs). (**a**) Schematic representation of the targeting strategy. The top shows the genomic locus of the NOTO gene together with the CRISPR target site in exon 3. Below is the targeting vector containing exon2, a NEO cassette, exon 3 linked via 2A to mCherry, and the 3′ UTR of the NOTO gene. The two bottom rows show a schematic of the targeted locus before and after removal of the NEO cassette. (**b**) Gel image of confirmation PCRs showing multiple hiPSC clones with successful NOTO-2A-mcherry insertion; (**c**) Morphology of the Noto-2A-mCherry reporter colonies of YK27-hiPSCs in free feeder layer conditions, which did not show mCherry fluorescence under the pluripotency state.

To generate a cell line that would allow easy visualization of NOTO expression levels and the differentiation stage, CRISPR/Cas9-based genome editing was used to introduce a NOTO-2A-mCherry targeting vector through homology-directed repair into the NOTO genomic locus in HDFa-YK27 hiPSCs using the strategy shown in Figure 1a. The targeting vector consisted of a LoxP-flanked NEO cassette, placed between exons 2 and 3 of the NOTO gene, the mCherry reporter sequence cloned after exon 3 of the NOTO gene upstream of the 3′UTR, and the sequence for the selectable marker rendering human cells resistant to DT-A (Diphtheria Toxin A) (Figure 1a). Importantly, we replaced the STOP codon and used the 2A system to link the open reading frame of the NOTO gene with the mCherry reporter sequence to obtain co-expression of NOTO and mCHERRY at equimolar levels [24]. We demonstrated the successful integration of the targeting vector into the NOTO gene through PCR in multiple clones (Figure 1b). After analysis by Sanger sequencing, no mutation was detected in the engineered hiPSC clones. To reduce clonal bias, we used one homozygous (#10.1) and one heterozygous clone (#14.3) for downstream analyses. Parental NOTO-2A-mCherry hiPSC clones (#10.1 and #14.3) were maintained in feeder-free culture conditions. During subculturing, these hiPSC colonies showed typical pluripotent cell morphology and did not express mCherry fluorescence in stem cell media conditions (Figure 1c). 

**Figure 2:**
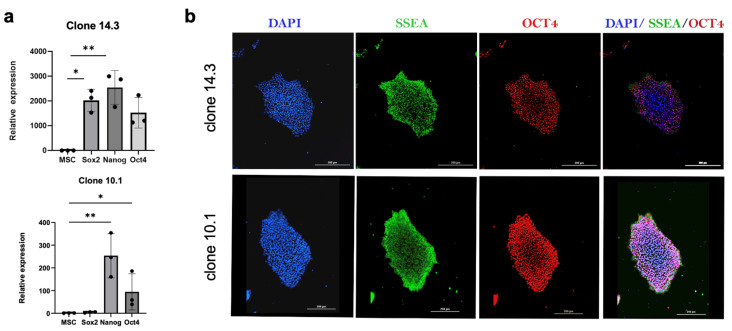
Noto-2A-mCherry iPSC clones maintain pluripotency. (**a**) RT- qPCR analysis of Noto-2A-mCherry-derived clones 14.3 and 10.1 showing expression of *NANOG, OCT4,* and *SOX2* stemness genes relative to expression in H9-MSCs. *GAPDH* was used as the housekeeping gene and internal control. * *p* ≤ 0.05; ** *p* ≤ 0.01. (**b**) Immunofluorescence staining for the stemness markers SSEA-4 and OCT4 in Noto-2A-mCherry clones 14.3 and 10.1. Scale bar = 200 μm.

We confirmed the pluripotency of the parental clones by analyzing the mRNA expression of stemness genes by RT- qPCR. Compared to expression in MSCs, we detected significant expression of pluripotency marker genes *OCT4, NANOG*, and *SOX2* in all selected clones (Figure 2a). Additionally, we performed immunofluorescent staining for stemness markers in these clones and our results showed robust protein expression of SSEA4 and OCT4 in parental clones, confirming pluripotent characteristics in newly generated heterozygous and homozygous NOTO-2A-reporter hiPSC lines (Figure 2b). 

### 3.2. Differentiation of hiPSCs into Notochordal-like Cells

**Figure 3:**
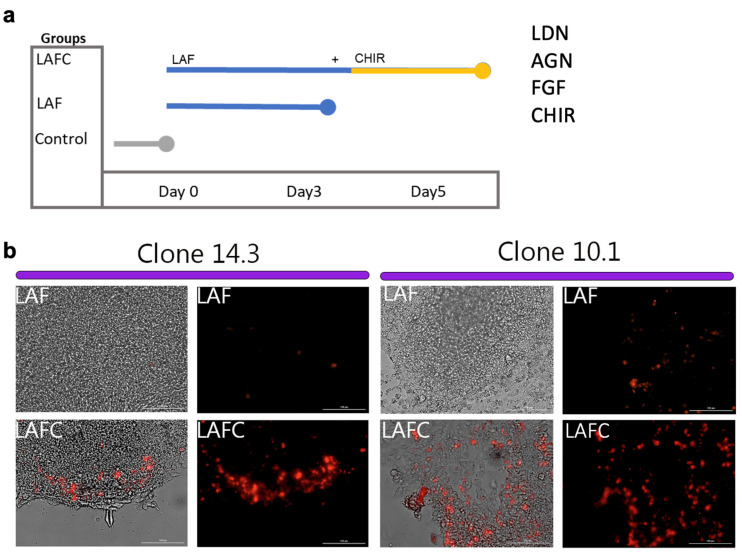
Derivation of notochordal cells (NCs) from YK-27-Noto-2A-mCherry hiPSC clones. (**a**) Schematic representation of the timeline and treatment strategy for the derivation of NCs using four factors (LAFC: **L**DN193189, **A**GN193109, b**F**GF, and **C**HIR99021); (**b**) Representative brightfield and fluorescent images of NOTO-2A-mCherry hiPSC clones 14.3 and 10.1 after 5 days of notochordal differentiation without the addition of CHIR99021 on day 3 (LAF) and with the addition of CHIR99021 on day 3 of differentiation (LAFC). Images were taken at 20× magnification; Scale bar = 100 μm.

To derive notochordal-like cells from hiPSCs, we adapted our previously reported stepwise differentiation protocol for hESCs with slight modifications [7]. We treated the cells with a cocktail of the BMP inhibitor **L**DN193189, the pan receptor inhibitor of retinoic acid signaling **A**GN193109, and basic **F**GF2 for three days (LAF treatment). On day 3, the GSKβ inhibitor **C**HIR99021 was added to the LAF cocktail and differentiation was continued until day 5 in the presence of the cocktail of all four factors (LAFC) (Figure 3a). Progression of notochordal commitment was monitored on day 3 and day 5 using mCherry fluorescence as a marker of *NOTO* expression. The initial commitment of hiPSCs towards the notochordal lineage was observed on the third day of LAF treatment. Microscopic visualization showed clusters of mCherry-positive cells; colonies did not show morphological changes and they continued to grow. In this intermediate notochord population (the LAF treatment group), mCherry fluorescence did not increase over time (Figure 3b). The addition of the small molecule **C**HIR99021 to the LAF cocktail elicited a robust increase in the number of NOTO mCherry-positive cell clusters on day 5 (Figure 3b). Microscopic visualization revealed that the LAFC cocktail gradually transformed the previously uniform, round-shaped colonies into two different areas; a compact area in the center that is surrounded by loose cells at the edges (Figure 3b and Figure S1). 

**Figure 4:**
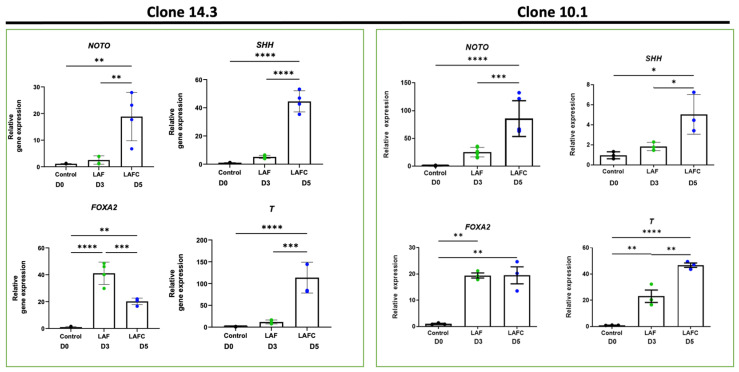
Increased expression of notochordal marker genes in YK-27-Noto-2A-mCherry hiPSC clones during notochordal differentiation. Gene expression of early notochord markers *NOTO, SHH, FOXA2,* and BRACHYURY (*T*) in clones 14.3 (**left**) and 10.1 (**right**) during differentiation, as detected by RT-qPCR. *GAPDH* gene served as the internal control and data are represented as expression relative to undifferentiated hiPSCs at day 0 (D0). All data are from at least three independent experiments and represented as mean ± SD. * *p* ≤ 0.05; ** *p* ≤ 0.01; *** *p* ≤ 0.001; **** *p* ≤ 0.0001.

At the transcriptional level, significant upregulation of *NOTO* expression, as well as the key inductors of node and notochordal fate *FOXA2*, *T, and SHH*, were detected in both clones (Figure 4). No significant differences were observed between clones with heterozygous or homozygous insertion of the transgene, indicating that the transgenic insertion does not affect *NOTO* expression or differentiation potential. In addition, we also assessed the expression of *FOXA1* and *CHRD* (*CHORDIN*), which are known to be expressed during initial notochord formation, as well as *ACAN* (*AGGRECAN)* and *SLIT2* as genes associated with the notochord and nucleus pulposus (Figure S2). We detected significant upregulation of these markers after 5 days of differentiation. Together, these data demonstrate that treatment of LAF followed by the CHIR component supports the molecular progression of NOTO-2A-mCherry hiPSC lines towards a notochordal fate. 

### 3.3. Global Transcriptomic Analysis during Notochordal Differentiation

**Figure 5:**
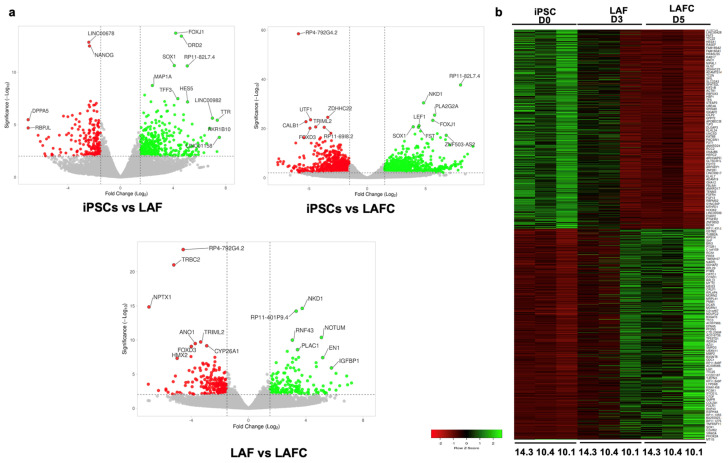
Transcriptome analysis during notochordal differentiation. Bulk RNA sequencing was performed on days 0, 3, and 5 of notochordal differentiation of 3 different clones. Differential gene expression analysis revealed distinct transcriptomic signatures between the three groups. (**a**) Volcano plots of genes differentially expressed (expression > 2-fold, false discovery rate [FDR] *p*-value < 0.05) between the different stages of notochordal differentiation. Upregulated genes are shown as green dots, downregulated genes as red dots (*n*  =  3 of each clone); (**b**) Heat map showing gene expression values of the most differentially expressed genes during notochordal differentiation on days 0, 3, and day 5 from three different hiPSC clones (#10.1, #10.4, and #14.3). Expression values for each gene (row) were normalized across all samples (columns) by Z score. Color key indicates the intensity associated with normalized expression values; Green color indicates higher expression and red color indicates lower expression of genes.

To study the key molecular players involved in differentiation from hiPSCs towards the notochordal lineage, we performed an unbiased high-throughput transcriptomic analysis. RNA was isolated on days 0, 3, and 5 of notochordal differentiation from three different clones and whole-transcriptome profiling was performed using bulk RNA sequencing. We first performed differential gene expression (DGE) analysis on days 3 (D3) and 5 (D5) in comparison to undifferentiated hiPSCs on day 0 (D0). Our analysis revealed that compared to the undifferentiated hiPSCs, 152 genes were upregulated and 116 genes were downregulated on day 3 during notochordal differentiation after treatment with LAF (FDR-corrected *p*-value < 0.05) (Figure 5a). On the other hand, LAFC treatment resulted in the upregulation of 1180 genes and downregulation of 1150 genes after 5 days of the differentiation process compared to undifferentiated hiPSCs (Figure 5a). DGE analyses between the LAF and LAFC treatment groups further showed that the addition of CHIR for 2 days resulted in the upregulation of 1028 genes and the downregulation of 1034 genes during notochordal differentiation (Figure 5a). Further analyses of differentially expressed genes during notochordal differentiation demonstrate gradual changes in the expression profiles of a large number of genes in all three clones, as shown in the heatmap (Figure 5b). 

**Figure 6:**
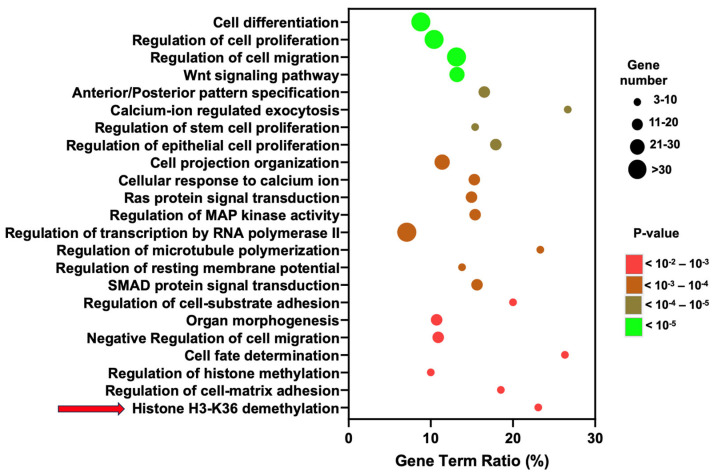
Gene ontology analysis of genes differentially expressed between hiPSCs and LAFC-treated cells at day 5 of notochordal differentiation. Dot plot of enriched genes categorized by biological processes. The horizontal axis represents the number of differentially expressed genes as the ratio of all genes within a GO term as “Gene Term Ratio”, while the vertical axis represents the enriched pathways. The color of the dots indicates the *p*-value and the size of the dots is relative to the number of differentially expressed genes within an identified biological process.

We next performed gene ontology (GO) enrichment analysis to annotate these differentially expressed genes by biological processes. At 5 days of notochordal differentiation, there was a significant enrichment of genes that play a role in several relevant biological processes in LAFC-treated cells, such as cell differentiation, cell migration, anterior–posterior pattern specification, cell fate determination, Wnt signaling, and calcium signaling (Figure 6, File S1). Furthermore, genes involved in histone methylation and demethylation pathways were enriched in LAFC treatment, suggesting the potential involvement of histone modification during notochordal differentiation (Figure 6, File S1).

### 3.4. Histone Modifiers as Regulators of hiPSC Differentiation into Notochordal Cells

**Figure 7:**
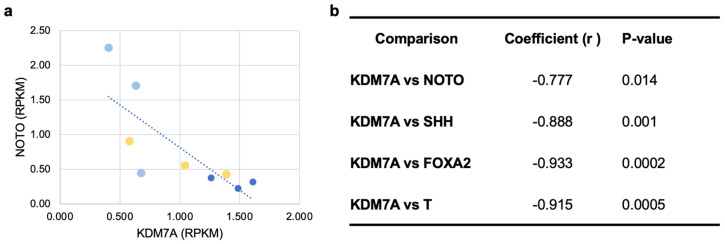
Negative correlation between expression levels of *KDM7A* and notochordal genes. Gene expression values of *KDM7A* during the course of notochordal differentiation on days 0, 3, and 5. (**a**) Linear regression analysis using Pearson correlation showing negative correlation of *KDM7A* with *NOTO* during notochordal differentiation. Data are represented as RPKM values from three independent clones. Dark blue dots—day 0, yellow dots—day 3, and light blue dots—day 5 of differentiation. (**b**) Table summarizing linear regression analysis using Pearson correlation showing inverse correlation of *KDM7A* expression with the expression of notochordal marker genes *SHH, NOTO, FOXA2,* and *T* based on RPKM values.

Histone H3-K36 demethylation was identified as one of the most significantly regulated pathways (arrow, Figure 6). This was driven by differential regulation of the histone demethylases *KDM7A*, *KDM4A*, and *KDM4C* (File S1). Members of the histone-lysine-demethylase families KDM4 and KDM7 remove, among others, mono-, di-, and trimethylation marks on lysine 36 on histone H3 [25]. To determine the potential role of KDMs during notochord differentiation, we performed a correlation analysis between the expression level of KDMs and notochordal marker genes using RPKM values. Interestingly, linear regression analyses showed an inverse correlation between the expression level of KDMs (*KDM7A, KDM4A,* and *KDM4C*) and notochordal genes such as *NOTO, FOXA2, SHH,* and *T* during the differentiation process (Figure 7 and Figure S3). These data suggest that inhibition of KDMs may promote the differentiation of hiPSCs into notochordal cells.

**Figure 8:**
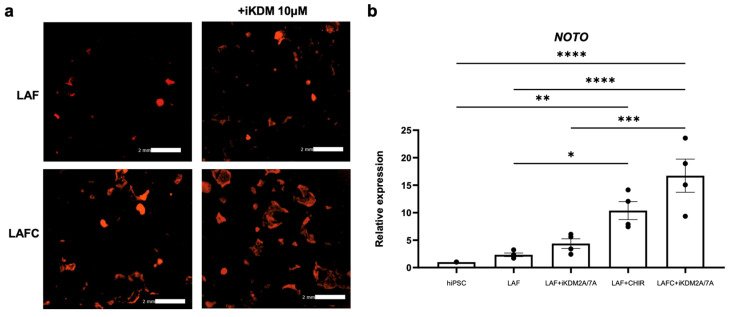
Inhibition of histone demethylases promotes the induction of hiPSC differentiation into notochordal-like cells. (**a**) Representative fluorescent images of NOTO-2A-mCherry-iPSCs during notochordal differentiation on day 3 of LAF or LAF + iKDM treatment and day 5 of LAFC or LAFC + iKDM treatment are shown. Scale bar 2 mm. (**b**) RT-qPCR analysis showing gene expression of *NOTO* in the 4 different treatment groups. *GAPDH* served as the internal control and data are represented as expression relative to undifferentiated hiPSCs at day 0 (D0). All data are represented as mean ± SD from four independent experiments. * *p* ≤ 0.05; ** *p* ≤ 0.01; *** *p* ≤ 0.001; **** *p* ≤ 0.0001.

To define the role of KDM7A in notochord differentiation, we investigated whether pharmacologic inhibition of KDMs promotes the lineage commitment of hiPSCs into notochordal cells. We used Daminozide, an inhibitor of KDM7A/B and KDM2A (referred to as iKDM), and added it to hiPSC cultures in the presence of LAF or LAFC and analyzed the effects of KDM2A/7A/7B inhibition on the notochordal differentiation of hiPSCs. We analyzed notochord differentiation using *NOTO* expression monitored by mCherry fluorescence as a marker. Our results showed that while LAF treatment induces weak mCherry expression, the addition of the KDM inhibitor to the LAF cocktail (LAF + iKDM) showed a robust induction of mCherry fluorescence, suggesting enhanced notochord differentiation already on day 3 (Figure 8a). Similarly, the addition of iKDM during the LAFC treatment further increased mCherry fluorescence, suggesting improved induction of notochordal differentiation. The increase in fluorescence was accompanied by some morphological changes. The colonies in the LAF + iKDM group were small, mCherry fluorescent cells were in clusters, and the fluorescence did not have any specific pattern. Interestingly, large and spread-out colonies were seen in the LAFC + iKDM treatment, with a central dense area with loosened and spread borders where mCherry cluster cells were observed. At the gene expression level, treatment with iKDM in both the LAF and LAFC groups induced the expression of *NOTO* compared to LAF or LAFC treatment alone (Figure 8b). However, this increase was not significant compared to LAF or LAFC treatment alone (LAF vs. LAF + iKDM *p* = 0.89; LAFC vs. LAFC + iKDM *p* = 0.08). These data further suggest that inhibition of KDM7A/B and KDM2A enhanced the notochordal differentiation of hiPSCs using our four-factor cocktail treatment.

## 4. Discussion

In this study, we utilized Noto-2A-mCherry hiPSCs to induce notochord differentiation using a four-factor approach that was previously established for hESCs by our lab [7]. This strategy relied on the known molecular mechanisms of notochord differentiation during development. Notochordal cell commitment is initiated by the expression of *FOXA2 and T*, which can control the expression of the transcription factor NOTO, specific to the node area [26]. Previous studies have shown that the node ^FoxA2+, T+, Noto+^ progenitor cells require Wnt signaling to support their axial extension to establish the notochord structure [27]. Consistent with this, the sequential treatment performed in the present study with LAF and LAFC cocktails aims to provide the landscape that restricts alternative fates (such as ectoderm or endoderm) and allows the induction of axial mesodermal signals represented by *FOXA2*, *T, NOTO,* and *SHH.* Activation of Wnt signaling through the addition of the small molecule CHIR99021 in the later phase of the protocol stabilizes and further advances the notochordal commitment, evidenced by the induction of notochord markers such as *FOXA2*, *T, FOXA1*, *CHRD*, *ACAN,* and *SLIT2*. In our previous study, we sorted Noto^+^ cells derived from hESCs, intending to expand the Noto^+^ cell population. However, a reduction in cell viability and slow proliferation were observed during subculturing, limiting the ability to obtain long-term cultures of pure Noto^+^ cells. These data demonstrated that maintaining a mixed population is more effective in the differentiation process and thus our protocol provides an effective strategy for notochordal derivation in hESCs and hiPSCs. 

Exploring the transcriptional profile of derived progenitor notochordal cells at specific commitment stages could help obtain homogeneity and identify key molecular players involved in notochord commitment. Therefore, we performed an unbiased transcriptome analysis at different stages of notochordal differentiation (Days 0, 3, and 5) to identify the molecular pathways that play a role in the differentiation process. Global transcriptome analysis using three independent hiPSC lines identified distinct gene expression profiles related to notochord commitment. Using GO term analysis, we showed the enrichment of differentially regulated genes belonging to various GO categories related to histone modification, suggesting the potential involvement of epigenetic regulation during notochordal differentiation. Interestingly, our results showed that expression of demethylation genes such as KDMs was downregulated upon treatment with a cocktail of LAF or LAFC factors, indicating that notochord differentiation of hiPSCs may be regulated by the levels of histone methylation. 

In the recent past, epigenetic regulation has been shown to play a key role in iPSC differentiation [28,29,30]. Histone methylation, particularly the trimethylated histone 3 lysine 4, 9, 27, and 36 (H3K4me3, H3K9me3, H3K27me3, and H3K36me3), are dominant epigenetic histone signatures [31]. One mechanism by which gene expression can be regulated at the epigenetic level is by modifying the extent of histone methylation. Previous studies have indicated that MSC differentiation is sensitive to epigenetic changes and that the degree of H3K9 acetylation and H3K4 trimethylation in MSCs increased, whereas the level of H3K9 trimethylation decreased during osteogenic differentiation [32,33]. Our global transcriptome analysis showed that *KDM7A, KDM4A*, and *KDM4C* are highly expressed during the pluripotent stage of hiPSCs and are downregulated during differentiation into notochordal cells (Figure 7 and Figure S3). Therefore, we hypothesized that repression of KDMs would enhance the commitment of hiPSCs into the notochordal lineage. Indeed, inhibition of KDM2A together with KDM7A and KDM7B through the addition of the small molecule Daminozide enhanced notochordal differentiation of hiPSCs in the presence of an LAF or LAFC cocktail. Therefore, the application of epigenetic regulators, such as inhibitors of histone-modifying enzymes, may be valuable in inducing the lineage commitment of hiPSCs [34]. Whether KDM inhibitors synergize with the effect of CHIR99021 to augment the activation of Wnt signaling and thus promote notochord differentiation or notochordal cell proliferation remains an open question. Therefore, it warrants further in-depth investigation to identify the consequences of KDM inhibition on Wnt signaling during notochordal differentiation. 

## 5. Conclusions

In conclusion, we have developed a model for enhanced derivation of notochord-like cells as potential NP precursors from hiPSCs. Our molecular characterization of lineage commitment and differentiation into notochordal cells revealed potential epigenetic control of the induced developmental progression. Focusing on a specific family of histone modifiers, we promoted notochordal cell derivation via inhibition of KDM family members in monolayer cultures. Future studies are warranted to promote the terminal maturation of these cells into NP structures, especially using 3D constructs. Moreover, we envision that these notochord-like progenitor cells will be suitable for NP tissue regeneration after modification. Our future studies will focus on their regenerative capabilities in the context of injury-induced or aging-induced inflammation during IDD progression. 

## Data Availability

The raw data supporting the conclusions of this article will be made available by the authors on request.

## Supplementary Materials

The following supporting information can be downloaded at: https://www.mdpi.com/article/10.3390/cells13171482/s1, Figure S1: Morphology of NOTO-2A-mCherry-hiPSCs at day 5 of notochordal differentiation; Figure S2: Embryonic notochordal cell marker expression during notochordal differentiation from human iPSCs (clones #10.1 and 14.3); Figure S3: Negative correlation between *KDM4A* and *KDM4C* with notochordal marker genes; File S1: GO term analysis.
