# Supplementary material for: Inhibition of KDM2/7 Promotes Notochordal Differentiation of hiPSCs
Source: Cells. 2024 Sep 4;13(17):1482. doi: 10.3390/cells13171482 (PMC11393929; doi:10.3390/cells13171482)
Supplement: Supplementary file 1 [file cells-13-01482-s001.zip › cells-3180021-supplementary/Diaz-Hernandez_appendix_A_rev.pdf]

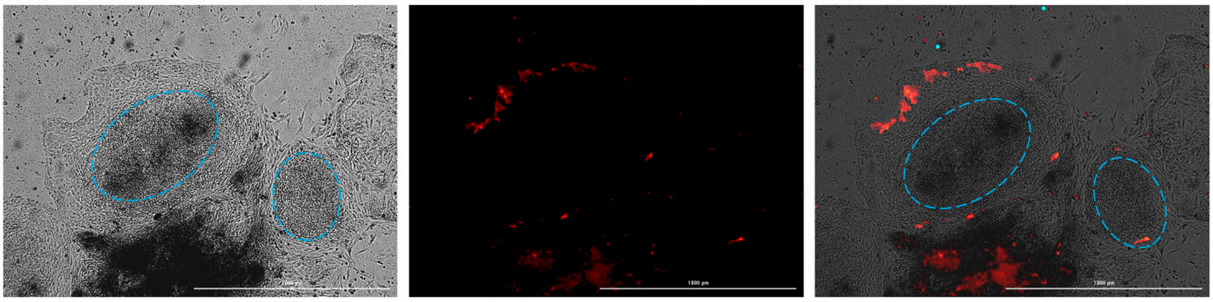

Figure S1: Morphology of NOTO-2A-mCherry-hiPSCs at day 5 of notochordal differentiation. Representative brightfield and fluorescent images of NOTO-2A-mCherry-hiPSC clone 10.1 at day 5 after treatment with LAF for 3 days and addition of CHIR99021 at day 3. Colonies show a compact area in the center (blue dotted circles), surrounded by loose cells at the edges. Images were taken at 10X magnification; Scale bar = 1000µm.

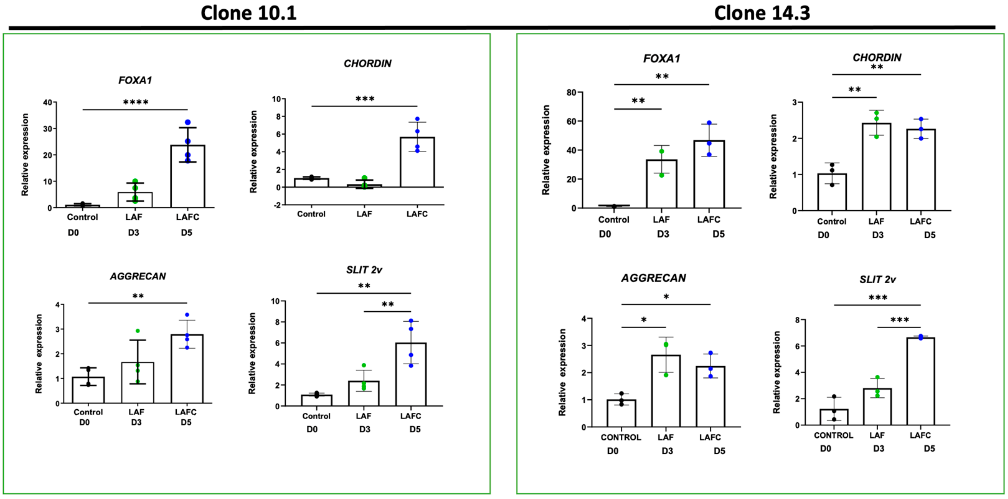

Figure S2: Embryonic notochordal cell marker expression during notochordal differentiation from human iPSCs (clone #10.1, 14.3). RT-qPCR analyses showed induced expression of *FOXA1, CHORDIN, SLIT2,* and *ACAN* in LAF and LAFC treatments of hiPSCs at day 3 (D3) and day 5 (D5) of notochordal differentiation. *GAPDH* gene served as internal control and data is represented as expression relative to undifferentiated hiPSC at day 0 (D0). All data are from three independent experiments and represented as mean±SD. * p≤0.05; ** p≤0.01; *** p≤0.001; **** p≤0.0001

a

Comparison	Coefficient (r)	P-value
**KDM4A vs NOTO**	-0.803	0.009
**KDM4A vs SHH**	-0.892	0.001
**KDM4A vs FOXA2**	-0.891	0.001
**KDM4A vs T**	-0.865	0.003

b

Comparison	Coefficient (r)	P-value
**KDM4C vs NOTO**	-0.697	0.037
**KDM4C vs SHH**	-0.832	0.005
**KDM4C vs FOXA2**	-0.939	0.0002
**KDM4C vs T**	-0.814	0.008

Figure S3: Negative correlation between *KDM4A* and *KDM4C* with notochordal marker genes. Linear regression analysis using Pearson correlation showing negative correlation of *KDM4A* (a) and *KDM4C* (b) with the expression of notochordal marker genes *SHH, NOTO, FOXA2,* and *T* based on RPKM values.
